# Lifestyle Habits and Comorbidities as Determinants of Quality of Life in Coronary Artery Disease: A Single-Center Prospective Study

**DOI:** 10.3390/jcm15062384

**Published:** 2026-03-20

**Authors:** Justyna Tokarewicz, Julia Kobylińska, Elżbieta Krajewska-Kułak, Barbara Jankowiak, Krystyna Klimaszewska, Michał Święczkowski, Sławomir Dobrzycki

**Affiliations:** 1 Department of Invasive Cardiology, Internal Medicine with CICU and Laboratory of Hemodynamics, Medical University of Białystok, Jana Kilińskiego 1, 15-089 Białystok, Poland; justynatokarewicz@gmail.com (J.T.);; 2 Department of Integrated Medical Care, Medical University of Białystok, Jana Kilińskiego 1, 15-089 Białystok, Poland

**Keywords:** coronary artery disease, quality of life, lifestyle factors, multimorbidity, prospective study

## Abstract

**Background**: Although survival in coronary artery disease (CAD) has improved with modern therapies, quality of life (QoL) remains an important clinical concern. Our study aimed to evaluate QoL, life satisfaction, and disease acceptance in CAD patients and to identify their clinical and lifestyle determinants. **Methods**: This single-center, prospective study included patients undergoing percutaneous coronary intervention for myocardial infarction (MI) or chronic coronary syndrome (CCS). QoL was assessed using validated questionnaires (WHOQOL-BREF, SWLS, AIS). Comparative analyses between the MI and CCS groups were performed, and the determinants of the outcomes were evaluated using regression models. **Results**: The study included 220 patients (110 MI and 110 CCS) with a median age of 64 years (IQR 54–70); 30% were women. The WHOQOL-BREF-assessed QoL was comparable between MI and CCS patients, whereas MI patients reported higher life satisfaction (SWLS 24 vs. 20, *p* = 0.003). Smoking was the strongest determinant of poorer QoL, associated with lower SWLS (β = −2.75; *p* < 0.001) and WHOQOL-BREF (β = −4.46; *p* = 0.014). Alcohol consumption (β = −6.22; *p* = 0.008), hypertension (β = −7.10; *p* < 0.001), and chronic obstructive pulmonary disease (β = −9.84; *p* < 0.001) were also independently associated with lower WHOQOL-BREF scores. Subgroup analyses showed heterogeneity between MI and CCS patients. **Conclusions**: QoL in CAD patients might be influenced more by lifestyle factors and multimorbidity than by CAD phenotype. Smoking, alcohol, and cardiopulmonary comorbidities might have the most consistent adverse associations with QoL. These findings highlight the potential importance of integrating lifestyle and comorbidity management to improve QoL and patient-reported outcomes in CAD care.

## 1. Introduction

Over the past century, myocardial infarction (MI) has evolved from a post-mortem pathological finding to a clinically recognized disease, progressing from conservative bed rest management to modern evidence-based therapies, including rapid reperfusion with percutaneous coronary intervention (PCI) [1]. Despite substantial global advances, ischemic heart disease, of which MI constitutes a significant component, accounted for an estimated 254.3 million prevalent cases, 9 million deaths, and 188.4 million disability-adjusted life years (DALYs) worldwide in 2021, remaining a major global health concern [2]. Data from the 2023 Global Burden of Cardiovascular Diseases and Risk Factors highlights an important insight, as approximately 79.6% of cardiovascular DALYs are attributable to modifiable risk factors such as hypertension (HT), elevated body mass index, tobacco use, or alcohol consumption, and are therefore preventable through effective risk factor management [3]. Furthermore, crude cardiovascular mortality is not expected to stay in the plateau phase between 2025 and 2050, but to increase by 73.4%, reaching 35.6 million deaths globally, with ischemic heart disease remaining the leading cause and accounting for approximately 20 million deaths in 2050, despite a 30.5% decline in age-standardized cardiovascular mortality rates, which reflects population aging, rather than worsening individual risk, as still posing a growing challenge to healthcare systems [4].

Improved survival following MI has increased interest in understanding patients’ quality of life (QoL) and long-term functional and psychosocial outcomes [5,6]. Health-related QoL (HRQoL) after MI is an important clinical outcome that captures patients’ perspectives on health status and provides a patient-centered measure of health, which can serve as an additional endpoint in evaluating care [7]. Depression and anxiety following MI likely represent stress-related neuropsychological responses to a life-threatening cardiovascular event, associated with concerns about prognosis and functional limitations. Psychological distress is increasingly recognized as both a contributor to and a consequence of cardiovascular disease [8]. Patients with coronary artery disease (CAD) frequently present with multiple comorbidities, which are associated with worse clinical outcomes and poorer QoL [9]. Overall, QoL in patients after MI is lower than that observed in other chronic conditions such as chronic obstructive pulmonary disease (COPD) and heart failure (HF) [10]. Moreover, behavioral factors such as low physical activity and prolonged sedentary behavior, as well as long-term exposure to air pollution, may further worsen post-MI QoL and adversely affect both physical and mental health [11,12].

CAD remains the leading cause of HF, significantly reducing patients’ QoL, increasing hospitalization rates, and elevating the risk of premature death [13]. Therefore, further investigation of its impact on QoL is essential. Despite guideline-directed medical therapy, HF progresses to an advanced stage in approximately 20–30% of patients, characterized by severe functional limitations and markedly reduced QoL. In such cases, a comprehensive approach, including pharmacological treatment, device-based therapies, heart transplantation, or palliative care, is required to optimize outcomes [14]. A multidisciplinary approach in patients with CAD improves long-term disease management and clinical outcomes, including QoL [13]. Studies indicate that team-based care reduces hospitalizations, increases patient satisfaction, and may be more cost-effective than usual care [15]. Integrating advanced practice nurses into cardiology teams improves care coordination and timely interventions. At the same time, nurse-led strategies such as telephone follow-up and remote monitoring can further enhance QoL, reduce hospital admissions, and support more effective HF management [16,17].

There is a broad consensus that advances in the treatment of MI should be accompanied by improvements in patients’ QoL, a complex, multidimensional construct. However, the previously published studies, including those involving large cohorts exceeding 9000 patients [7,10], have typically relied on a single assessment tool, such as the EuroQoL-5D [6,7,10] and the Short Form-36 Health Survey [12]. Consequently, the multidimensional nature of QoL in patients with CAD is frequently assessed using a single measurement instrument, which may not fully capture its different domains or reflect the complex interplay between clinical, psychosocial, and lifestyle-related factors. To address this limitation, the present study adopts an integrated multi-scale approach by incorporating three complementary instruments assessing distinct dimensions of patient-reported outcomes. Our study evaluates three key dimensions of QoL-patients’ adaptation to health limitations—the Acceptance of Illness Scale (AIS), self-perceived QoL (WHOQOL-BREF), and the Satisfaction with Life Scale (SWLS). These outcomes were assessed in a prospective and well-balanced cohort of patients with MI and chronic coronary syndrome (CCS) undergoing PCI, representing two major clinical phenotypes of CAD. Importantly, the study also examines the role of modifiable lifestyle factors and coexisting comorbidities, including cardiopulmonary diseases, which frequently accompany CAD and may substantially influence patient-reported outcomes. Our study puts a particular focus on modifiable lifestyle factors such as smoking, alcohol consumption, and body mass index, which may remain insufficiently controlled and may significantly influence post-cardiac event QoL. By simultaneously evaluating multiple dimensions of QoL and their associations with lifestyle factors and multimorbidity across two distinct CAD phenotypes, this study provides a more comprehensive perspective on the determinants of patient-reported outcomes in contemporary CAD populations.

The primary objective of the study was to assess the QoL, disease acceptance, and life satisfaction in patients with CAD. The secondary objectives were to evaluate the impact of key modifiable lifestyle factors and comorbidities on these outcomes and to compare both the outcomes and their determinants between patients with MI and CCS.

## 2. Methods

### 2.1. Study Design

This was a single-center, prospective, questionnaire-based study conducted at the Department of Invasive Cardiology at the Medical University of Bialystok, Poland. The study was designed and reported in accordance with the STROBE guidelines (Supplementary Table S1) and was conducted in compliance with the Declaration of Helsinki.

All patients admitted to the department between 19 December 2023 and 30 June 2025 were screened for eligibility. The inclusion criteria were: age ≥ 18 years; hospital admission for PCI due to MI or CCS; and provision of written informed consent to participate in the study. The exclusion criteria included withdrawal of consent or lack of consent to participate in the study, as well as more than 10% of questionnaire data missing.

The MI group included patients diagnosed with type 1 MI according to the Fourth Universal Definition of Myocardial Infarction and treated with PCI during the index hospitalization. In contrast, the CCS group comprised patients with CAD with angiographically and/or functionally significant coronary artery stenosis who were qualified for PCI [18].

During the study period, 5713 patients were assessed for eligibility; 230 consecutive patients meeting the inclusion criteria were enrolled. All enrolled participants completed a set of anonymous, self-administered patient-reported questionnaires assessing baseline characteristics, lifestyle habits, and QoL, using the AIS, SWLS, and WHOQOL-BREF.

Following data validation and quality control performed independently by two reviewers, 10 patients were excluded from further analysis due to incomplete data. The final analytical cohort comprised 220 CAD patients, including 110 MI patients and 110 CCS patients, and was included in the final analyses.

**Figure 1:**
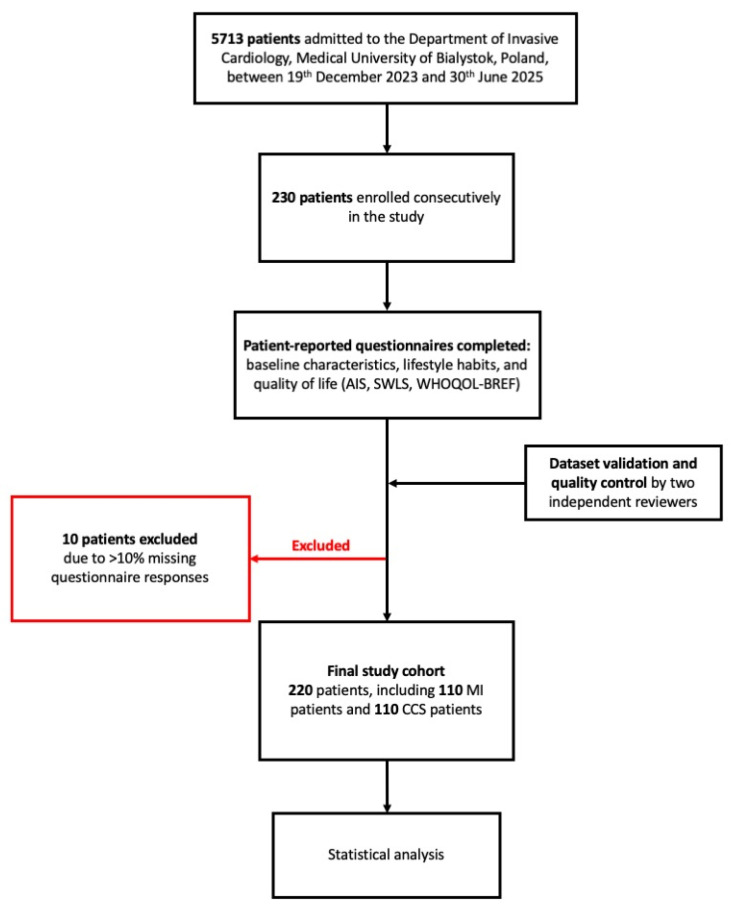
Study design. Abbreviations: AIS, Acceptance of Illness Scale; CCS, chronic coronary syndrome; MI, myocardial infarction; SWLS, Satisfaction With Life Scale; and WHOQOL-BREF, World Health Organization Quality of Life—BREF.

The study protocol was reviewed and approved by the Bioethics Committee of the Medical University of Bialystok, Poland (approval number: APK.002.549.2023). All participants provided written informed consent before inclusion in the study. The study design flowchart is presented as Figure 1.

### 2.2. Data Collection

The questionnaires were self-administered and paper-based. Patients completed them once during hospitalization, typically on the second day of their hospital stay, in a quiet hospital setting that allowed for independent completion of the forms. Participants were instructed to answer all questions based on their current perceptions and experiences and were given sufficient time to complete the questionnaires without time pressure.

In older patients, or when understanding specific questions was difficult, a trained interviewer provided clarification on the wording or meaning of the items, without suggesting or prompting any answers. The interviewer’s role was limited strictly to explaining the content of the questions to ensure proper understanding, while the patients independently selected their responses. This procedure was applied to minimize the potential response bias and to ensure the reliability and completeness of the collected data.

QoL was assessed using three standardized research instruments with validated Polish versions. It was evaluated using the WHOQOL-BREF questionnaire developed by the World Health Organization in 1996 [19]. The instrument consists of 26 items, enabling the assessment of QoL across four domains: physical, psychological, social, and environmental. The assessed areas include, among others, the experience of somatic complaints, functioning in interpersonal relationships, psychological well-being, living conditions, and socioeconomic security. It is assumed that higher scores reflect a better subjective assessment of QoL. WHOQOL-BREF results were analyzed separately for each of the four domains, and the obtained values were transformed to a 0–100 scale in accordance with World Health Organization guidelines. The level of adaptation to illness was assessed using the AIS, derived from Felton and colleagues’ concept [20]. The scale comprises eight statements referring to the subjective perception of limitations resulting from illness, including loss of independence, dependence on others, and reduced self-esteem. The total score, ranging from 8 to 40 points, constitutes a measure of illness acceptance, with higher values indicating better psychological adjustment to the disease. Subjective life satisfaction was assessed using the SWLS, which was developed by Diener and colleagues [21]. The instrument consists of five statements rated on a seven-point response scale, yielding a total score ranging from 5 to 35 points. Higher scores indicate greater global life satisfaction. The internal consistency of the instruments in the present sample was high (Cronbach’s α = 0.904 for AIS, 0.868 for SWLS, and 0.924 for WHOQOL-BREF). Moreover, the Polish versions of these instruments have previously demonstrated good psychometric properties in validation studies [22,23]. A summary of all instruments used is presented in Supplementary Table S2.

### 2.3. Statistical Analysis

Continuous variables were assessed for normality using the Shapiro–Wilk test. Due to non-normal distributions, between-group comparisons of continuous variables were performed using the Mann–Whitney U test. In contrast, categorical variables were analyzed using the χ^2^ test or Fisher’s exact test, as appropriate, depending on the expected cell counts. Continuous data are presented as medians (Me) with interquartile ranges (1Q–3Q), and categorical variables as counts (N) and percentages (%).

To identify factors associated with patient-reported outcomes, linear regression analyses were applied. AIS, SWLS, and WHOQOL-BREF were analyzed as continuous dependent variables. Independent variables included age, sex, smoking status, alcohol use, and BMI, selected a priori based on clinical relevance. Univariate linear regression models were first used to assess crude associations, followed by multivariable models, including all predictors simultaneously to estimate the adjusted effects and account for potential confounding. Model assumptions were evaluated for all linear regression models by assessing the linearity, residual distribution, homoscedasticity, and multicollinearity. Linearity was examined visually, using residual-versus-fitted plots. Multicollinearity was assessed using variance inflation factors; all were below commonly accepted thresholds, indicating no relevant collinearity among predictors. Homoscedasticity was evaluated using the Breusch–Pagan test, and the residual normality was examined using the Shapiro–Wilk test. Because heteroscedasticity and deviations from normality in the residuals were observed in several models, heteroscedasticity-consistent robust standard errors (HC3) were used to ensure valid statistical inference.

All analyses were conducted in the entire cohort and repeated separately in MI and CCS subgroups to explore potential differences between the two groups. No imputation methods were used for the missing values. Regression results are reported as coefficients with 95% confidence intervals (95% CI). Statistical significance was defined as a two-sided *p*-value < 0.05. All statistical analyses were performed using STATA version 18 (StataCorp LLC, College Station, TX, USA).

## 3. Results

### 3.1. Cohort Characteristics

In our study, we included 220 CAD patients, with 110 in the MI group and 110 in the CCS group. The MI and CCS groups were well-balanced in age and sex. Men predominated in both groups (75.5% in MI vs. 64.5% in CCS, *p* = 0.077), and the median age was comparable (61 [54–69] vs. 65 [54–70] years, *p* = 0.182). In the overall cohort, HT (69.5%), diabetes mellitus (24.1%), and COPD (5%) were the most prevalent comorbidities. Patients with CCS had a higher prevalence of previous MI (35.5% vs. 0.9%, *p* < 0.001) and HF (7.3% vs. 0.9%, *p* = 0.041) than those with MI, while other comorbidities were comparable between the groups. No significant differences were observed in the lifestyle factors, anthropometric parameters, BMI, or obesity prevalence.

Regarding patient-reported outcomes, life satisfaction, assessed by the SWLS, was significantly higher in the MI group (24 [17–25] vs. 20 [15–24], *p* = 0.003). Acceptance of illness measured by AIS did not differ significantly between groups (33 [26.25–37.75] vs. 32 [26–34], *p* = 0.454). Similarly, overall QoL and all WHOQOL-BREF domains were comparable between MI and CCS patients. Table 1 presents baseline demographic and clinical characteristics, as well as patient-reported outcomes, for the MI and CCS groups.

### 3.2. Cohort Analyses

In the whole cohort, age was not significantly associated with AIS, SWLS, or WHOQOL-BREF. No significant sex-related differences were observed for AIS or SWLS, although women reported higher WHOQOL-BREF scores than men [99.0 (87.25–103.0) vs. 93.0 (80.0–101.0), *p* = 0.006].

Smoking status was strongly associated with QoL outcomes, with current smokers showing significantly lower SWLS [20.0 (15.0–24.5) vs. 24.0 (19.0–26.0), *p* < 0.001] and WHOQOL-BREF scores [91.0 (80.0–100.0) vs. 98.0 (90.5–103.5), *p* < 0.001] compared with non-smokers. At the same time, AIS did not differ between groups. Alcohol use was also related to poorer outcomes, as alcohol drinkers had lower SWLS [18.0 (15.0–24.5) vs. 21.0 (17.0–25.0), *p* = 0.038] and lower WHOQOL-BREF scores [83.0 (74.0–95.0) vs. 97.0 (87.0–102.0), *p* < 0.001]. Obesity was associated with lower AIS [28.0 (26.0–34.0) vs. 33.0 (27.0–37.75), *p* = 0.029] and WHOQOL-BREF scores [90.0 (78.0–100.0) vs. 97.0 (86.0–102.0), *p* = 0.005].

A history of previous MI was linked to lower AIS [28.5 (26.0–34.0) vs. 33.0 (27.0–38.0), *p* = 0.027] and WHOQOL-BREF scores [89.5 (78.75–97.0) vs. 96.0 (84.0–102.0), *p* = 0.019], while the difference in SWLS did not reach statistical significance. HT was associated with significantly lower SWLS [20.0 (15.0–25.0) vs. 22.0 (19.0–27.0), *p* = 0.002] and WHOQOL-BREF scores [91.0 (79.0–101.0) vs. 100.0 (92.0–105.0), *p* < 0.001], whereas AIS did not differ between hypertensive and normotensive patients. HF was not associated with AIS, SWLS, or WHOQOL-BREF. Diabetes mellitus was associated with significantly lower WHOQOL-BREF scores [88.0 (78.0–100.0) vs. 97.0 (86.0–102.0), *p* = 0.002], while AIS and SWLS did not differ between diabetic and non-diabetic patients. COPD patients reported lower SWLS [15.0 (13.0–16.0) vs. 21.0 (16.0–25.0), *p* = 0.001] and WHOQOL-BREF scores [81.0 (73.0–89.0) vs. 95.0 (83.25–102.0), *p* = 0.005]. Table 2 summarizes the QoL, acceptance of illness, and life satisfaction outcomes across demographic, lifestyle, and comorbidity subgroups in the whole cohort. Data on WHOQOL-BREF domain scores in the whole cohort are presented in Supplementary Table S3.

### 3.3. Subgroup Analyses

In the MI group, women had lower AIS than men [27.0 (24.0–33.0) vs. 32.0 (25.5–38.0), *p* = 0.032]. Smokers and alcohol users reported lower SWLS [21.5 (15.0–25.0) vs. 25.0 (19.0–26.25), *p* = 0.023 and 18.5 (15.0–23.25) vs. 25.0 (18.75–25.0), *p* = 0.011, respectively] and lower WHOQOL-BREF [92.0 (79.25–101.75) vs. 100.0 (90.0–104.5), *p* = 0.021 and 82.5 (71.0–94.75) vs. 98.0 (87.0–102.5), *p* = 0.007, respectively]. MI patients with HT also had lower SWLS [22.0 (15.0–25.0) vs. 25.0 (21.0–27.0), *p* = 0.006] and lower WHOQOL-BREF [91.0 (78.0–102.0) vs. 100.0 (94.0–104.5), *p* = 0.002].

In the CCS group, women reported higher WHOQOL-BREF than men [99.0 (89.0–103.0) vs. 91.0 (80.0–100.0), *p* = 0.007]. Smokers had lower SWLS [17.0 (15.0–20.0) vs. 21.0 (18.0–25.0), *p* < 0.001] and lower WHOQOL-BREF [88.0 (80.75–100.0) vs. 97.5 (90.75–103.0), *p* = 0.007]. Alcohol use was associated with lower WHOQOL-BREF [83.0 (75.0–97.5) vs. 95.0 (86.0–102.0), *p* = 0.011]. Obese patients showed significantly lower AIS [25.0 (22.0–30.25) vs. 32.0 (26.0–33.0), *p* = 0.001] and lower WHOQOL-BREF [83.0 (75.75–94.75) vs. 96.5 (85.5–102.0), *p* = 0.004] than non-obese patients. Lower WHOQOL-BREF was also observed in CCS patients with HT [91.0 (80.0–100.0) vs. 98.0 (91.0–105.0), *p* = 0.002] and diabetes mellitus [85.0 (77.5–94.5) vs. 96.0 (84.0–102.0), *p* = 0.012]. CCS patients with COPD reported significantly lower SWLS [13.0 (10.0–13.0) vs. 20.0 (15.0–24.0), *p* = 0.006]. Table 3 presents the QoL, acceptance of illness, and life satisfaction outcomes across the demographic, lifestyle, and comorbidity subgroups in the MI and CCS cohorts. Data on WHOQOL-BREF domain scores in CCS and MI patients are summarized in Supplementary Table S4.

### 3.4. Univariate Regression Analyses

In the whole cohort, age was associated with lower WHOQOL-BREF (β = −5.58; *p* = 0.002), whereas sex showed no significant associations. Among lifestyle factors, smoking and alcohol consumption were associated with lower SWLS (β = −3.12; *p* < 0.001 and β = −2.04; *p* = 0.045, respectively) and WHOQOL-BREF (β = −6.41; *p* < 0.001 and β = −8.57; *p* < 0.001, respectively). Higher BMI was linked to lower WHOQOL-BREF (β = −0.76; *p* = 0.002). HT was linked to all QoL measures (AIS: β = −2.10; *p* = 0.033, SWLS: β = −2.77; *p* < 0.001, and WHOQOL-BREF: β = −9.59; *p* < 0.001). COPD was associated with lower SWLS (β = −6.02; *p* < 0.001) and lower WHOQOL-BREF (β = −12.47; *p* = 0.002), whereas diabetes mellitus was associated only with lower WHOQOL-BREF (β = −7.38; *p* < 0.001). A history of MI was associated with lower AIS (β = −2.52; *p* = 0.024) and lower WHOQOL-BREF (β = −5.38; *p* = 0.03), indicating a broader adverse impact on both disease acceptance and global QoL.

Subgroup analyses showed both shared and distinct patterns. In MI patients, age was positively associated with AIS (β = 4.04; *p* = 0.02). Lifestyle factors remained relevant in this group, with smoking and alcohol consumption being associated with both lower SWLS and WHOQOL-BREF. HT continued to show adverse associations with SWLS (β = −3.33; *p* = 0.003) and WHOQOL-BREF (β = −10.76; *p* < 0.001), whereas diabetes mellitus remained associated with lower WHOQOL-BREF (β = −7.03; *p* = 0.037). In CCS patients, higher BMI was associated with lower AIS (β = −0.49; *p* = 0.003) and WHOQOL-BREF (β = −1.10; *p* = 0.002). Smoking was related to lower SWLS (β = −3.74; *p* < 0.001) and WHOQOL-BREF (β = −6.25; *p* = 0.011), while alcohol consumption was only associated with lower WHOQOL-BREF (β = −7.28; *p* = 0.028). HT was linked to lower AIS (β = −2.80; *p* = 0.039) and WHOQOL-BREF (β = −8.38; *p* = 0.001), while COPD was associated with lower SWLS (β = −8.75; *p* < 0.001) and WHOQOL-BREF (β = −15.69; *p* = 0.026). Diabetes mellitus was related to lower WHOQOL-BREF (β = −7.68; *p* = 0.010). HF was not significantly associated with any QoL measure in the whole cohort or in either subgroup. The detailed results of univariate regression are presented in Table 4.

### 3.5. Multivariable Regression Analyses

In multivariable regression analyses, only a limited set of variables retained independent associations with QoL in the whole cohort. Smoking remained related to lower SWLS (β = −2.747; *p* < 0.001) and lower WHOQOL-BREF (β = −4.457; *p* = 0.014). Alcohol consumption was associated with lower WHOQOL-BREF (β = −6.217; *p* = 0.008). Among the cardiometabolic factors, HT remained related to lower SWLS (β = −2.295; *p* = 0.012) and lower WHOQOL-BREF (β = −7.100; *p* < 0.001), while COPD was associated with lower SWLS (β = −5.628; *p* < 0.001) and lower WHOQOL-BREF (β = −9.835; *p* < 0.001). No independent associations were observed between age, sex, BMI, diabetes mellitus, or previous MI and any QoL measure in the whole cohort, and no variable remained independently associated with AIS after adjustment.

In the MI subgroup, age remained independently associated with higher AIS (β = 4.392; *p* = 0.011). Alcohol consumption was associated with lower SWLS (β = −3.555; *p* = 0.023) and WHOQOL-BREF (β = −8.933; *p* = 0.012). The cardiometabolic burden remained relevant in this group: HT and COPD were associated with lower SWLS (β = −3.212; *p* = 0.012 and β = −3.574; *p* = 0.032, respectively) and lower WHOQOL-BREF (β = −9.117; *p* < 0.001 and β = −7.313; *p* = 0.031, respectively). No independent effects of smoking, BMI, and diabetes mellitus were observed in MI patients. In the CCS subgroup, a different pattern emerged. Higher BMI remained independently associated with lower AIS (β = −0.476; *p* = 0.006). Smoking was independently related to lower SWLS (β = −3.385; *p* < 0.001). COPD remained associated with lower SWLS (β = −7.794; *p* = 0.002) and WHOQOL-BREF (β = −12.451; *p* = 0.034). No independent associations were observed for alcohol consumption, HT, diabetes mellitus, age, sex, or previous MI with any QoL measure in CCS patients. The detailed results of multivariable regression are presented in Table 5.

## 4. Discussion

Our study assessed HRQoL in two subgroups: patients revascularized due to MI or CCS. The present study demonstrated that QoL between the two subgroups does not differ markedly; however, patients in the acute MI phase reported significantly higher SWLS scores than those in the CCS group. QoL in those two subgroups seems to be determined by patients’ lifestyle. Inadequately controlled modifiable factors that may have had the strongest negative impact on QoL are smoking, alcohol consumption, and obesity. Moreover, the burden of comorbidities, especially HT and COPD, is a potentially important determinant of poorer QoL in both analyzed subgroups.

### 4.1. Contextualizing Our Findings

Differences between MI and CCS patients were observed in a study comparing QoL among three patient groups (CCS, HF, and MI), using a different measurement instrument—the MacNew questionnaire—which suggests that the significantly poorer HRQoL observed in patients with CCS compared to MI patients may be explained by persistent chest discomfort and related physical limitations [24]. The only significant difference in our study between the CCS and MI groups was a higher subjective SWLS score in patients with MI, which may be attributable to a response shift phenomenon, an adjustment of internal standards for evaluating HRQoL after an adverse health event, which can reflect successful coping or intervention, but may also influence HRQoL measurements and affect whether long-term assessments show the actual clinical status or are masked by a post-survival relief effect [25]. Meanwhile, no significant variations were observed in AIS, which we assume reflects that the acceptance of illness is a gradual process, with prolonged three-stage adaptation as its component, which is probably shaped by personality, social support, and individual resources [5], rather than being influenced by rapid changes such as those observed in the short-term response shift phenomenon. There is still no consensus on the impact of successful PCI on QoL. The available studies report a wide spectrum of outcomes, ranging from unfavorable trends characterized by increasing levels of anxiety compared with the baseline [26], through to patterns of transient improvement, where a favorable decline in mental stress is observed before hospital discharge but the prevalence of depressive and anxiety symptoms gradually increases within one year after PCI [27], to findings in which an initial post-procedural improvement in QoL is followed by deterioration at 6 and 12 months in specific patient subgroups [28]. This pattern may be explained by illusion theory, whereby the sudden and marked improvement in QoL reflects a transient euphoric and overly optimistic perception immediately after successful treatment, which diminishes as patients become more aware of their long-term condition, which may partly account for our findings [28]. In contrast, Dimagli et al. reported beneficial improvements in QoL over time following PCI [29].

We hypothesize that these paradoxical and contrary findings may be due to differences in adaptation and may be caused by situations when acute-phase patients benefit from intensive medical support while chronic-phase patients face long-term limitations and persistent concerns that can reduce QoL, highlighting the crucial role of cardiac rehabilitation and psychological support in preserving well-being after the event. There is substantial evidence that structured cardiac rehabilitation following MI improves both the physical and psychological aspects of QoL [30,31]. Cardiac rehabilitation programs, which were developed for the prevention and management of CAD, include ‘physical part’- medical treatment, as well as ‘psychical part,’ supporting the emotional and social functioning of each patient [30]. Patients participating in hospital-based rehabilitation show significant improvement in both physical and mental component scores compared with non-rehabilitated patients [30]. During the ambulatory phase of cardiac rehabilitation, even in the absence of dedicated psychological resources, an overall improvement in mental health is demonstrated; nonetheless, approximately 20% of patients continue to manifest clinically relevant depression or anxiety symptoms upon completion of the program, highlighting the need for targeted psychological interventions [30]. It is still concerning that the participation rates of patients with chronic vascular disease among hospital-based cardiac rehabilitation programs remain low, varying between 10% and 35% of eligible patients [32].

### 4.2. Role of Modifiable Risk Factors

In our analysis, modifiable risk factors, including smoking, alcohol consumption, and obesity, emerged as stronger determinants of QoL than the disease category itself. We suggest that this may be explained by the theory that pro-health behaviors influence patients’ daily functioning, developing both physical and psychological well-being. For instance, recent evidence indicates that tobacco smoking is associated with lower HRQoL, particularly among heavy smokers, while former smokers also exhibit poorer outcomes compared with non-smokers [33]. Another study conducted among patients with CAD examined associations between lifestyle modifications and HRQoL, demonstrating that lifestyle modification was strongly associated with improved QoL, with higher observations not only among never-smokers but also among individuals who quit smoking [34]. In contrast to studies demonstrating that higher BMI is consistently associated with reduced QoL [6], De Smedt et al. show that changes in BMI were not significantly related to QoL, indicating that healthier behaviors such as physical activity and dietary improvement, rather than weight loss per se, are key determinants of QoL [34]. Inconsistency between our findings and those of De Smedt et al. may reflect methodological differences, including more detailed behavioral stratification compared with BMI-based classification in our study. Moreover, alcohol dependence and heavy alcohol use are associated with significantly lower HRQoL [35]. Alcohol may also alter the pharmacokinetics and pharmacodynamics of commonly prescribed medications [36], potentially compromising therapeutic efficacy and symptom control and thereby further reducing QoL.

The progressive aging of the population is associated with an increasing burden of comorbid conditions, representing an inevitable challenge for healthcare systems, with recent large-scale population-based data from England indicating a comorbidity prevalence of 68.2% among individuals aged 80 years and older [37]. According to the Global Burden of Disease Study, approximately three in five global deaths are attributed to the four major non-communicable diseases: cardiovascular disease, cancer, chronic respiratory diseases, and diabetes [38]. It is therefore unsurprising that the present study identifies a notable association between diminished QoL and comorbid conditions, including HT, COPD, and diabetes. Numerous studies have demonstrated an association between HT and reduced QoL. Two large meta-analyses confirmed its negative impact on HRQoL, affecting both mental and physical domains [39,40]. Although among hypertensive patients, the issue of QoL is not entirely straightforward, some studies have reported that patients who do not adhere to medication present with higher HRQoL, which might be attributed to the negative impact of the perceived necessity of taking medications [41]; on the other hand, results from a Greek multicenter study indicate that suboptimal medication adherence is prevalent among patients with HT and dyslipidemia, and is independently associated with reduced HRQoL [42]. Interestingly, a study examining three trajectories of QoL in patients following MI showed that those who did not improve, whose QoL declined between hospitalization and 12 months of follow-up, often suffer from diabetes and COPD [7]. In a Korean study assessing HRQoL in patients during the acute phase of MI, diabetes was associated with poorer HRQoL outcomes [43]. Similarly, patients with diabetes have been shown to report significantly lower HRQoL than non-diabetic controls, with women exhibiting greater impairments across several domains [44]. In addition, because cardiovascular disease and COPD share common risk factors, they frequently coexist; Wills et al. reported that COPD patients with concomitant cardiovascular disease already experience significantly reduced HRQoL at early disease stages [45]. Specifically studying patients after MI, those with COPD exhibit worse HRQoL and impaired physical functioning up to one year after the event compared with those without COPD, highlighting COPD as an important factor influencing post-MI recovery [46].

The previously noted differences in studies [26,27,28,29] examining changes in HRQoL over time, together with evidence from longitudinal analysis demonstrating that while overall HRQoL may improve, a substantial proportion of patients with CAD experience significantly declines after 5 years [47], underscore the need for further longitudinal research to investigate the long-term trajectories of HRQoL and to identify the patient subgroups and domains that are most responsive to clinical interventions.

Healthy lifestyle, risk factor modification, and medication adherence are vital for preventing mortality and recurrent events in individuals with CAD [48]. Studies tend to confirm that lifestyle changes have a positive impact on QoL [34,49]; moreover, the substantial economic burden imposed on European healthcare systems by chronic vascular diseases is estimated to be 282 billion euros annually [50]; therefore, the importance of low-cost, scalable interventions has arisen. We believe that future research should focus on evaluating and optimizing lifestyle modifications as a cost-effective strategy to improve patient outcomes and QoL.

Among patients with heart disease, depression and anxiety disorders are highly prevalent and persistent, playing a significant role in cardiovascular disease progression and standing as independent risk factors [27]. Also, depressive symptoms have a negative impact on subsequent HRQoL among patients with CAD [51].

### 4.3. Special Populations

Increasing attention has recently been given to patients with ischemia with no obstructive coronary artery disease (INOCA), who constitute a significant and often underrecognized clinical population. Studies indicate that more than 50% of individuals with stable angina and approximately 10–15% of those referred for ACS have no obstructive coronary lesions, highlighting the need for careful assessment of HRQoL in this group [52]. The health status of patients with suspected INOCA—including angina frequency, physical limitations, and QoL during the 12 months following elective angiography for chest pain—has been shown to be comparable to that of patients with stable angina and obstructive CAD [53]. Moreover, both physical and mental QoL indices at the 12-month follow-up remain worse than those of healthy individuals, underscoring that improving health status through effective angina management is a key therapeutic goal, as angina frequency is strongly associated with patients’ QoL [53]. As endothelial dysfunction may be an underlying mechanism in INOCA, Xiao et al. investigated whether changes in endothelial function predict QoL. Although causality cannot be established, improvements in flow-mediated dilation over one year were associated with higher QoL scores, providing a potential direction for future research [54].

### 4.4. Clinical and Supportive Perspectives

Critically, attention should be directed toward the clinical implications and potential interventions, as routine assessment of HRQoL enables early identification of high-risk patients and facilitates personalized management strategies that may reduce adverse outcomes and improve long-term well-being [55]. Moreover, it is important to recognize that women after MI may experience lower HRQoL trajectories compared with men, highlighting the need to consider sex-specific differences when evaluating patients and planning treatment strategies [56].

If HRQoL assessment is not incorporated into standard care, opportunities to identify patients who may benefit from targeted interventions may be missed, potentially leading to preventable readmissions and increased healthcare costs [55]. For example, poor baseline HRQoL in patients undergoing PCI has been shown to be strongly associated with higher one-year mortality and major adverse cardiovascular events independently of the established risk factors [55].

The growing recognition of the clinical relevance of HRQoL raises important questions about how these outcomes can be systematically assessed in routine clinical practice [57]. Such an approach requires solutions that enable regular data collection without imposing excessive burden on patients or the healthcare system. Evidence suggests that electronic patient-reported outcome monitoring systems may represent a promising tool, as they can improve patient–physician communication, enhance the clarity of treatment explanations, and increase patients’ knowledge about their cardiac condition [57].

Therefore, once this high-risk cohort is identified, targeted interventions such as structured exercise-based cardiac rehabilitation programs can be implemented, as evidence demonstrates that these programs significantly improve HRQoL compared with the usual care for patients undergoing coronary procedures [58].

### 4.5. Study Strengths

The strengths of our study include a well-characterized patient cohort, composed of two clinically relevant and well-balanced groups of patients, those with MI and CCS, which enabled a direct comparison between two major clinical phenotypes of CAD. Additionally, the use of three validated instruments to assess QoL and related psychosocial outcomes, a practice rarely reported in the literature, enhances the reliability and robustness of the outcomes, and the collection of detailed data on lifestyle habits, comorbidities, and risk factors enables a comprehensive evaluation of the modifiable determinants of QoL.

### 4.6. Study Limitations

Our study has several limitations that should be acknowledged. It is a single-center study, conducted in a tertiary cardiology center, which may limit the generalizability of the findings to other healthcare settings and populations. In addition, the study population comprised hospitalized CAD patients undergoing PCI, which may introduce selection bias and limit the results’ applicability to the broader CAD population. The relatively moderate sample size should also be considered when interpreting the results. The questionnaire-based, self-reported nature of the data introduces the potential for recall bias, response bias, and misestimation of symptom severity, and the fact that QoL was assessed during hospitalization may have further influenced patient responses, due to the acute clinical context. Another limitation of the study is the lack of a disease-specific instrument, such as the Seattle Angina Questionnaire, to assess angina-related symptoms. Furthermore, the cross-sectional design precludes causal inference and does not allow for the assessment of longitudinal changes in QoL. Additionally, detailed information on other relevant psychosocial factors, such as depression, anxiety, and social support, was not available, which may have influenced the observed outcomes. Finally, not all potentially relevant clinical and socioeconomic variables could be fully accounted for.

## 5. Conclusions

In this cohort of CAD patients, QoL appeared to be more strongly associated with lifestyle factors and multimorbidity than with the clinical form of CAD itself. Unhealthy behaviors, particularly smoking and alcohol consumption, together with cardiopulmonary comorbidities such as HT and COPD, may represent the most consistent adverse associations with QoL across multiple measures. These findings suggest that comprehensive management strategies that address both lifestyle factors and the comorbidity burden may play an important role in improving patient-reported outcomes in CAD patients. From a clinical perspective, these findings emphasize that strategies targeting lifestyle factors and the comorbidity burden may be relevant not only for prognosis but also for improving QoL in CAD patients. Further studies are warranted to confirm these observations and to explore their implications for clinical practice.

## Figures and Tables

**Table 1:** Baseline characteristics and patient-reported outcomes in the analyzed cohort.

Variable	All	MI	CCS	*p*-Value
Patients	220	110	110	N/A
**Baseline characteristics**				
Male, N (%)	154 (70%)	83 (75.5%)	71 (64.5%)	0.077
Age, Me (1Q–3Q)	64 (54–70)	61 (54–69)	65 (54–70)	0.182
Previous MI	40 (18.2%)	1 (0.9%)	39 (35.5%)	<0.001
Hypertension	153 (69.5%)	76 (69.1%)	77 (70%)	1
Heart failure	9 (4.1%)	1 (0.9%)	8 (7.3%)	0.041
Diabetes mellitus	53 (24.1%)	26 (23.6%)	27 (24.5%)	1
COPD	11 (5%)	6 (5.5%)	5 (4.5%)	1
**Marital status**				
Married, N (%)	164 (74.5%)	75 (68.2%)	89 (80.9%)	0.03
Divorced, N (%)	21 (9.5%)	13 (11.8%)	8 (7.3%)	0.251
Single, N (%)	12 (5.5%)	7 (6.4%)	5 (4.5%)	0.553
Widow/er, N (%)	22 (10%)	15 (13.6%)	7 (6.4%)	0.072
**Education level**				
Primary education, N (%)	58 (26.4%)	29 (26.4%)	29 (26.4%)	1
Vocational education, N (%)	45 (20.5%)	20 (18.2%)	25 (22.7%)	0.403
Secondary education, N (%)	65 (29.5%)	33 (30%)	32 (29.1%)	0.883
Higher education, N (%)	51 (23.2%)	28 (25.5%)	23 (20.9%)	0.424
**Occupational status**				
Rural residence, N (%)	88 (40%)	41 (37.3%)	47 (42.7%)	0.41
Physical worker, N (%)	71 (32.6%)	46 (41.8%)	25 (23.1%)	0.003
Retired/pensioner, N (%)	108 (49.5%)	46 (41.8%)	62 (57.4%)	0.021
Intellectual work, N (%)	34 (15.6%)	15 (13.6%)	19 (17.6%)	0.421
**Lifestyle factors**				
A current smoker, N (%)	123 (55.9%)	66 (60%)	57 (51.8%)	0.222
An alcohol user, N (%)	51 (23.2%)	22 (20%)	29 (26.4%)	0.263
**Anthropometric parameters**				
Height [m], Me (1Q–3Q)	1.73 (1.69–1.78)	1.74 (1.7–1.78)	1.72 (1.68–1.78)	0.776
Weight [kg], Me (1Q–3Q)	81 (72–91)	82 (74–90)	81 (70–91)	0.585
BMI [kg/m^2^], Me (1Q–3Q)	27.34 (24.87–29.74)	27.47 (25–29.63)	27.01 (24.52–29.74)	0.26
Obesity, N (%)	54 (24.5%)	26 (23.6%)	28 (25.5%)	0.754
**Quality of life scales**				
AIS, Me (1Q–3Q)	32 (26–36)	33 (26.25–37.75)	32 (26–34)	0.454
SWLS, Me (1Q–3Q)	21 (15–25)	24 (17–25)	20 (15–24)	0.003
WHOQOL-BREF, Me (1Q–3Q)	95 (82–102)	95 (84–102)	94 (82–101)	0.372
WHOQOL-BREF Somatic domain (0–100), Me (1Q–3Q)	57.14 (46.43–64.29)	60.71 (42.86–64.29)	57.14 (49.11–65.18)	0.969
WHOQOL-BREF Psychological domain (0–100), Me (1Q–3Q)	66.67 (54.17–75)	66.67 (58.33–79.17)	66.67 (54.17–75)	0.261
WHOQOL-BREF Social domain (0–100), Me (1Q–3Q)	66.67 (58.33–75)	66.67 (58.33–75)	66.67 (58.33–75)	0.606
WHOQOL-BREF Environmental domain (0–100), Me (1Q–3Q)	75 (59.38–78.12)	75 (59.38–78.12)	68.75 (56.25–75)	0.135

**Abbreviations:** AIS, Acceptance of Illness Scale; BMI, body mass index; CCS, chronic coronary syndrome; COPD, chronic obstructive pulmonary disease; kg, kilogram; m, meter; Me, median; MI, myocardial infarction; N, number of subjects; N/A, not applicable; SWLS, Satisfaction With Life Scale; WHO, World Health Organization; WHOQOL-BREF, World Health Organization Quality of Life—BREF; and Q, quartile.

**Table 2:** Subgroup comparison for the entire study cohort.

Whole Cohort, N = 220			
**Age**	**<65 years old**	**≥65 years old**	***p*-value**
AIS, Me (1Q–3Q)	33 (27–37)	31 (26–35.75)	0.223
SWLS, Me (1Q–3Q)	21 (16.25–25)	20 (15–25)	0.526
WHOQOL-BREF, Me (1Q–3Q)	97 (84–102)	93.5 (81–102)	0.402
**Sex**	**Female**	**Male**	
AIS, Me (1Q–3Q)	32.5 (26–38)	32 (27–33)	0.324
SWLS, Me (1Q–3Q)	20 (16–25)	21 (15–25)	0.683
WHOQOL-BREF, Me (1Q–3Q)	99 (87.25–103)	93 (80–101)	0.006
**Smoking**	**Non-smokers**	**Current smokers**	
AIS, Me (1Q–3Q)	32 (27–35)	32 (26–37.5)	0.744
SWLS, Me (1Q–3Q)	24 (19–26)	20 (15–24.5)	<0.001
WHOQOL-BREF, Me (1Q–3Q)	98 (90.5–103.5)	91 (80–100)	<0.001
**Alcohol use**	**Non-drinkers**	**Drinkers**	
AIS, Me (1Q–3Q)	33 (27–36)	29 (25.5–35)	0.062
SWLS, Me (1Q–3Q)	21 (17–25)	18 (15–24.5)	0.038
WHOQOL-BREF, Me (1Q–3Q)	97 (87–102)	83 (74–95)	<0.001
**BMI**	**<30 kg/m^2^**	**≥30 kg/m^2^**	
AIS, Me (1Q–3Q)	33 (27–37.75)	28 (26–34)	0.029
SWLS, Me (1Q–3Q)	21 (16–25)	20 (15–25)	0.401
WHOQOL-BREF, Me (1Q–3Q)	97 (86–102)	90 (78–100)	0.005
**Previous MI**	**no**	**yes**	
AIS, Me (1Q–3Q)	33 (27–38)	28.5 (26–34)	0.027
SWLS, Me (1Q–3Q)	21 (16–25)	20 (15–24.25)	0.092
WHOQOL-BREF, Me (1Q–3Q)	96 (84–102)	89.5 (78.75–97)	0.019
**Hypertension**	**no**	**yes**	
AIS, Me (1Q–3Q)	32 (29–38.5)	32 (26–35)	0.11
SWLS, Me (1Q–3Q)	22 (19–27)	20 (15–25)	0.002
WHOQOL-BREF, Me (1Q–3Q)	100 (92–105)	91 (79–101)	<0.001
**Heart failure**	**no**	**yes**	
AIS, Me (1Q–3Q)	32 (26–36)	28 (27–33)	0.783
SWLS, Me (1Q–3Q)	21 (15–25)	21 (20–29)	0.442
WHOQOL-BREF, Me (1Q–3Q)	95 (82–102)	95 (91–110)	0.54
**Diabetes mellitus**	**no**	**yes**	
AIS, Me (1Q–3Q)	32 (26–35.5)	32 (27–37)	0.688
SWLS, Me (1Q–3Q)	21 (16–25)	20 (15–25)	0.812
WHOQOL-BREF, Me (1Q–3Q)	97 (86–102)	88 (78–100)	0.002
**COPD**	**no**	**yes**	
AIS, Me (1Q–3Q)	32 (27–36)	27 (23–35.5)	0.224
SWLS, Me (1Q–3Q)	21 (16–25)	15 (13–16)	0.001
WHOQOL-BREF, Me (1Q–3Q)	95 (83.25–102)	81 (73–89)	0.005

**Abbreviations:** AIS, Acceptance and Action Scale; BMI, body mass index; COPD, chronic obstructive pulmonary disease; kg, kilogram; m, meter; Me, median; MI, myocardial infarction; N, number of subjects; Q, quartile; SWLS, Satisfaction With Life Scale; and WHOQOL-BREF, World Health Organization Quality of Life—BREF.

**Table 3:** Subgroup comparison in MI and CCS cohorts.

Variable	MI, N = 110			CCS, N = 110		
**Age**	**<65 years old**	**≥65 years old**	***p*-value**	**<65 years old**	**≥65 years old**	***p*-value**
AIS, Me (1Q–3Q)	32 (26–37.75)	30 (24.75–35)	0.095	32 (25–33)	29.5 (24–32)	0.32
SWLS, Me (1Q–3Q)	22 (17.25–25)	25 (17–27)	0.38	20 (16–25)	19 (15–22)	0.209
WHOQOL-BREF, Me (1Q–3Q)	95 (84.5–101)	98 (81.5–103)	0.787	97 (83.5–102)	91 (81–100)	0.175
**Sex**	**Female**	**Male**	***p*-value**	**Female**	**Male**	***p*-value**
AIS, Me (1Q–3Q)	27 (24–33)	32 (25.5–38)	0.032	32 (29–32)	28 (24–33)	0.085
SWLS, Me (1Q–3Q)	22 (16–29)	24 (17–25)	0.642	19 (16–22)	20 (15–25)	0.814
WHOQOL-BREF, Me (1Q–3Q)	100 (86.5–104.5)	95 (81–102)	0.165	99 (89–103)	91 (80–100)	0.007
**Smoking**	**Non-smokers**	**Current smokers**	***p*-value**	**Non-smokers**	**Current smokers**	***p*-value**
AIS, Me (1Q–3Q)	32 (25–35.25)	32 (25–37)	0.774	32 (25–33)	30 (24–32)	0.399
SWLS, Me (1Q–3Q)	25 (19–26.25)	21.5 (15–25)	0.023	21 (18–25)	17 (15–20)	<0.001
WHOQOL-BREF, Me (1Q–3Q)	100 (90–104.5)	92 (79.25–101.75)	0.021	97.5 (90.75–103)	88 (80.75–100)	0.007
**Alcohol use**	**Non-drinkers**	**Drinkers**	***p*-value**	**Non-drinkers**	**Drinkers**	***p*-value**
AIS, Me (1Q–3Q)	32 (25–36)	29.5 (24–34.5)	0.287	32 (25–33)	26 (24–32)	0.121
SWLS, Me (1Q–3Q)	25 (18.75–25)	18.5 (15–23.25)	0.011	20 (15–23)	18(15–25)	0.82
WHOQOL-BREF, Me (1Q–3Q)	98 (87–102.5)	82.5 (71–94.75)	0.007	95 (86–102)	83 (75–97.5)	0.011
**BMI**	**<30 kg/m^2^**	**≥30 kg/m^2^**	***p*-value**	**<30 kg/m^2^**	**≥30 kg/m^2^**	***p*-value**
AIS, Me (1Q–3Q)	32 (24.75–36)	29 (25–34.75)	0.662	32 (26–33)	25 (22–30.25)	0.001
SWLS, Me (1Q–3Q)	24 (17–25)	21 (15.5–25)	0.444	19.5 (15–22)	20 (13–25)	0.702
WHOQOL-BREF, Me (1Q–3Q)	98 (86–102)	93 (78–102)	0.252	96.5 (85.5–102)	83 (75.75–94.75)	0.004
**Previous MI**	**no**	**yes**	***p*-value**	**no**	**yes**	***p*-value**
AIS, Me (1Q–3Q)	N/A	N/A	N/A	32 (27.5–36)	29 (26–34)	0.073
SWLS, Me (1Q–3Q)	N/A	N/A	N/A	20 (15.5–23.5)	20 (15–24.5)	0.647
WHOQOL-BREF, Me (1Q–3Q)	N/A	N/A	N/A	96 (83.5–102)	91 (81–97)	0.059
**Hypertension**	**no**	**yes**	***p*-value**	**no**	**yes**	***p*-value**
AIS, Me (1Q–3Q)	32 (29–38)	33 (26–37)	0.558	32 (30–40)	31 (26–34)	0.106
SWLS, Me (1Q–3Q)	25 (21–27)	22 (15–25)	0.006	20 (17–25)	20 (15–23)	0.155
WHOQOL-BREF, Me (1Q–3Q)	100 (94–104)	91(78–102)	0.002	98 (91–105)	91 (80–100)	0.002
**Heart failure**	**no**	**yes**	***p*-value**	**no**	**yes**	***p*-value**
AIS, Me (1Q–3Q)	N/A	N/A	N/A	32 (26–34)	28 (26.75–30)	0.446
SWLS, Me (1Q–3Q)	N/A	N/A	N/A	20 (15–23.75)	20.5 (18.5–26)	0.517
WHOQOL-BREF, Me (1Q–3Q)	N/A	N/A	N/A	93.5 (82–101)	94.5 (88–101)	0.796
**Diabetes mellitus**	**no**	**yes**	***p*-value**	**no**	**yes**	***p*-value**
AIS, Me (1Q–3Q)	33 (26–37.25)	33 (27–37.75)	0.813	32 (26–34)	32 (27.5–35.5)	0.741
SWLS, Me (1Q–3Q)	24 (18–25)	21 (15–26)	0.705	19 (15–24.5)	20 (15–23.5)	0.72
WHOQOL-BREF, Me (1Q–3Q)	98 (87–102)	88 (78–101.5)	0.06	96 (84–102)	85 (77.5–94.5)	0.012
**COPD**	**no**	**yes**	***p*-value**	**no**	**yes**	***p*-value**
AIS, Me (1Q–3Q)	33 (27–38)	27 (25.25–31.75)	0.228	32 (26–34)	27 (21–38)	0.594
SWLS, Me (1Q–3Q)	24 (17.75–25)	16 (15–20.75)	0.065	20 (15–24)	13 (10–13)	0.006
WHOQOL-BREF, Me (1Q–3Q)	97 (86–102.5)	80 (75.75–91.75)	0.075	94 (82–101)	81 (63–84)	0.064

**Abbreviations:** AIS, Acceptance and Action Scale; BMI, body mass index; COPD, chronic obstructive pulmonary disease; kg, kilogram; m, meter; Me, median; MI, myocardial infarction; N, number of subjects; N/A, not applicable; Q, quartile; SWLS, Satisfaction With Life Scale; and WHOQOL-BREF, World Health Organization Quality of Life—BREF.

**Table 4:** Univariate predictors of QoL outcomes.

	Whole Cohort			MI			CCS		
	Coefficient	95% CI	*p*-Value	Coefficient	95% CI	*p*-Value	Coefficient	95% CI	*p*-Value
**AIS**									
Age	0.805	−1.12, 2.731	0.412	4.044	0.648, 7.439	0.02	−1.856	−4.068, 0.357	0.1
Male	−0.05	−0.131, 0.03	0.22	−0.094	−0.216, 0.028	0.131	−0.003	−0.116, 0.109	0.953
Smoking	0.011	−1.868, 1.89	0.991	0.773	−2.184, 3.729	0.608	−0.763	−3.191, 1.664	0.538
Alcohol	−1.929	−4.194, 0.336	0.095	−1.773	−5.461, 1.916	0.346	−2.036	−4.948, 0.876	0.171
BMI	−0.158	−0.416, 0.1	0.231	0.15	−0.268, 0.568	0.481	−0.49	−0.815, −0.165	0.003
Hypertension	−2.097	−4.028, −0.166	0.033	−1.403	−4.273, 1.466	0.338	−2.797	−5.447, −0.146	0.039
COPD	−2.794	−8.052, 2.464	0.298	−3.423	−10.142, 3.296	0.318	−2.076	−12.118, 7.965	0.685
Diabetes mellitus	0.155	−2.147, 2.456	0.895	0.043	−3.565, 3.651	0.981	0.27	−2.732, 3.271	0.86
Previous MI	−2.517	−4.695, −0.338	0.024	N/A	N/A	N/A	−2.435	−4.83, −0.04	0.046
Heart failure	−0.114	−4.407, 4.18	0.959	9.853	−8.123, 27.829	0.283	−1.328	−5.289, 2.632	0.511
**SWLS**									
Age	0.052	−1.672, 1.775	0.953	−0.692	−3.663, 2.279	0.648	0.144	−1.918, 2.206	0.891
Male	−0.026	−0.09, 0.039	0.437	−0.013	−0.098, 0.073	0.775	−0.023	−0.115, 0.07	0.632
Smoking	−3.119	−4.627, −1.611	<0.001	−2.886	−5.143, −0.629	0.012	−3.736	−5.663, −1.81	<0.001
Alcohol	−2.042	−4.038, −0.045	0.045	−3.943	−7.093, −0.794	0.014	−0.144	−2.761, 2.472	0.914
BMI	−0.08	−0.277, 0.117	0.428	−0.1	−0.389, 0.189	0.497	−0.09	−0.379, 0.199	0.54
Hypertension	−2.768	−4.414, −1.122	<0.001	−3.329	−5.511, −1.146	0.003	−2.152	−4.562, 0.259	0.08
COPD	−6.019	−9.314, −2.724	<0.001	−3.913	−7.615, −0.212	0.038	−8.752	−12.698, −4.807	<0.001
Diabetes mellitus	−0.354	−2.305, 1.597	0.722	−0.816	−3.701, 2.069	0.579	0.149	−2.526, 2.823	0.913
Previous MI	−1.897	−3.844, 0.049	0.056	N/A	N/A	N/A	−0.502	−2.663, 1.659	0.649
Heart failure	1.547	−3.432, 6.527	0.543	8.376	−9.58, 26.332	0.361	1.694	−3.517, 6.905	0.524
**WHOQOL-BREF**									
Age	−5.584	−9.103, −2.065	0.002	−4.517	−10.36, 1.326	0.13	−6.919	−11.444, −2.393	0.003
Male	−0.12	−0.283, 0.042	0.147	−0.161	−0.382, 0.061	0.154	−0.069	−0.316, 0.179	0.587
Smoking	−6.408	−9.983, −2.832	<0.001	−6.898	−12.234, −1.562	0.011	−6.25	−11.077, −1.423	0.011
Alcohol	−8.566	−13.436, −3.696	<0.001	−9.921	−17.553, −2.288	0.011	−7.284	−13.775, −0.793	0.028
BMI	−0.755	−1.225, −0.285	0.002	−0.472	−1.122, 0.179	0.155	−1.096	−1.797, −0.395	0.002
Hypertension	−9.588	−13.05, −6.127	<0.001	−10.758	−15.503, −6.014	<0.001	−8.376	−13.504, −3.248	0.001
COPD	−12.474	−20.535, −4.413	0.002	−9.887	−20.624, 0.851	0.071	−15.685	−29.541, −1.83	0.026
Diabetes mellitus	−7.378	−11.719, −3.036	<0.001	−7.033	−13.645, −0.421	0.037	−7.679	−13.528, −1.83	0.01
Previous MI	−5.382	−10.255, −0.51	0.03	N/A	N/A	N/A	−4.526	−9.72, 0.668	0.088
Heart failure	3.074	−9.794, 15.941	0.64	20.176	−356.147, 396.499	0.916	1.475	−12.325, 15.275	0.834

**Abbreviations:** AIS, Acceptance of Illness Scale; BMI, body mass index; CCS, chronic coronary syndrome; COPD, chronic obstructive pulmonary disease; MI, myocardial infarction; N/A, not applicable; SWLS, Satisfaction With Life Scale; and WHOQOL-BREF, World Health Organization Quality of Life—BREF.

**Table 5:** Multivariable predictors of QoL outcomes.

	Whole Cohort			MI			CCS		
	Coefficient	95% CI	*p*-Value	Coefficient	95% CI	*p*-Value	Coefficient	95% CI	*p*-Value
**AIS**									
Age	1.842	−0.169, 3.854	0.073	4.392	1.014, 7.771	0.011	−0.734	−3.597, 2.13	0.615
Male	−0.032	−0.123, 0.059	0.485	−0.064	−0.194, 0.066	0.335	0.053	−0.08, 0.187	0.435
Smoking	0.378	−1.546, 2.302	0.7	0.484	−2.42, 3.389	0.744	−0.277	−2.819, 2.264	0.831
Alcohol	−2.204	−4.566, 0.158	0.067	−3.059	−6.899, 0.781	0.118	−0.512	−3.886, 2.861	0.766
BMI	−0.162	−0.444, 0.12	0.261	0.02	−0.421, 0.462	0.928	−0.476	−0.816, −0.136	0.006
Hypertension	−2.079	−4.191, 0.033	0.054	−1.38	−4.399, 1.639	0.37	−2.079	−5.176, 1.018	0.188
COPD	−2.668	−7.798, 2.461	0.308	−3.642	−9.873, 2.588	0.252	−0.34	−9.352, 8.671	0.941
Diabetes mellitus	1.626	−1.125, 4.377	0.247	1.558	−2.787, 5.904	0.482	2.379	−0.972, 5.73	0.164
Previous MI	−1.843	−4.178, 0.491	0.122	N/A	N/A	N/A	−1.763	−4.589, 1.064	0.222
**SWLS**									
Age	1.275	−0.453, 3.003	0.148	0.58	−2.527, 3.686	0.715	1.023	−1.277, 3.322	0.384
Male	0.001	−0.068, 0.07	0.983	0.003	−0.101, 0.108	0.95	0.002	−0.1, 0.103	0.974
Smoking	−2.747	−4.297, −1.197	<0.001	−1.886	−4.366, 0.594	0.136	−3.385	−5.366, −1.404	<0.001
Alcohol	−1.571	−3.479, 0.337	0.107	−3.555	−6.623, −0.487	0.023	−0.039	−2.656, 2.579	0.977
BMI	−0.01	−0.214, 0.194	0.925	−0.093	−0.41, 0.224	0.563	0.003	−0.295, 0.301	0.985
Hypertension	−2.295	−4.095, −0.496	0.012	−3.212	−5.719, −0.705	0.012	−1.788	−4.457, 0.88	0.189
COPD	−5.628	−8.684, −2.572	<0.001	−3.574	−6.85, −0.299	0.032	−7.794	−12.718, −2.87	0.002
Diabetes mellitus	0.445	−1.486, 2.376	0.651	0.567	−2.581, 3.716	0.724	0.464	−2.12, 3.048	0.725
Previous MI	−1.584	−3.462, 0.295	0.098	N/A	N/A	N/A	−0.296	−2.406, 1.815	0.784
**WHOQOL-BREF**									
Age	−1.085	−4.449, 2.279	0.527	−1.133	−6.971, 4.705	0.704	−2.69	−7.25, 1.871	0.248
Male	0.014	−0.142, 0.171	0.858	−0.081	−0.315, 0.152	0.495	0.135	−0.107, 0.377	0.274
Smoking	−4.457	−8.001, −0.913	0.014	−4.663	−10.08, 0.754	0.092	−4.425	−9.369, 0.518	0.079
Alcohol	−6.217	−10.836, −1.599	0.008	−8.933	−15.927, −1.938	0.012	−3.472	−10.148, 3.205	0.308
BMI	−0.349	−0.798, 0.099	0.127	−0.331	−0.982, 0.319	0.318	−0.573	−1.256, 0.111	0.101
Hypertension	−7.1	−10.832, −3.367	<0.001	−9.117	−14.177, −4.057	<0.001	−5.025	−10.751, 0.702	0.085
COPD	−9.835	−15.608, −4.063	<0.001	−7.313	−13.973, −0.653	0.031	−12.451	−23.985, −0.917	0.034
Diabetes mellitus	−3.965	−8.61, 0.679	0.094	−1.914	−8.598, 4.771	0.575	−4.735	−11.435, 1.964	0.166
Previous MI	−3.008	−7.512, 1.496	0.191	N/A	N/A	N/A	−1.863	−6.846, 3.121	0.464

**Abbreviations:** AIS, Acceptance of Illness Scale; BMI, body mass index; CCS, chronic coronary syndrome; COPD, chronic obstructive pulmonary disease; MI, myocardial infarction; SWLS, Satisfaction With Life Scale; and WHOQOL-BREF, World Health Organization Quality of Life—BREF.

## Data Availability

Data could be shared by the corresponding author upon reasonable request.

## Supplementary Materials

The following supporting information can be downloaded at: https://www.mdpi.com/article/10.3390/jcm15062384/s1, Supplementary Table S1: STROBE checklist. Supplementary Table S2: Comparative characteristics of the WHOQOL-BREF, AIS, and SWLS scales. Supplementary Table S3: Domains of the WHOQOL-BREF scale in the entire cohort. Supplementary Table S4: Domains of the WHOQOL-BREF scale in subgroups.
