# Supplementary material for: Lifestyle Habits and Comorbidities as Determinants of Quality of Life in Coronary Artery Disease: A Single-Center Prospective Study
Source: J Clin Med. 2026 Mar 20;15(6):2384. doi: 10.3390/jcm15062384 (PMC13027064; doi:10.3390/jcm15062384)
Supplement: Supplementary file 1 [file jcm-15-02384-s001.zip › jcm-4183914-supplementary.pdf]

Title: Lifestyle Habits and Comorbidities as Determinants of Quality of Life in Coronary Artery Disease: A Single-Center Prospective Study

Authors: Justyna Tokarewicz, Julia Kobylińska, Elżbieta Krajewska-Kułak, Barbara Jankowiak, Krystyna Klimaszewska, Michał Święczkowski, Sławomir Dobrzycki

Supplementary materials

Supplementary Table S1. STROBE checklist.

	Item No	Recommendation	Page number
**Title and abstract**	1	(*a*) Indicate the study's design with a commonly used term in the title or the abstract	1
		(*b*) Provide in the abstract an informative and balanced summary of what was done and what was found	1
**Introduction**			
Background/rationale	2	Explain the scientific background and rationale for the investigation being reported	2-3
Objectives	3	State specific objectives, including any prespecified hypotheses	53
**Methods**			
Study design	4	Present key elements of study design early in the paper	3-4, Figure 1
Setting	5	Describe the setting, locations, and relevant dates, including periods of recruitment, exposure, follow-up, and data collection	3-5
Participants	6	(*a*) *Cohort study*—Give the eligibility criteria, and the sources and methods of selection of participants. Describe methods of follow-up<br>*Case-control study*—Give the eligibility criteria, and the sources and methods of case ascertainment and control selection. Give the rationale for the choice of cases and controls	3-5

		Cross-sectional study—Give the eligibility criteria, and the sources and methods of selection of participants	
		(*b*) *Cohort study*—For matched studies, give matching criteria and number of exposed and unexposed<br>*Case-control study*—For matched studies, give matching criteria and the number of controls per case	
Variables	7	Clearly define all outcomes, exposures, predictors, potential confounders, and effect modifiers. Give diagnostic criteria, if applicable	3-5
Data sources/ measurement	8*	For each variable of interest, give sources of data and details of methods of assessment (measurement). Describe comparability of assessment methods if there is more than one group	5-6
Bias	9	Describe any efforts to address potential sources of bias	5-6
Study size	10	Explain how the study size was arrived at	3
Quantitative variables	11	Explain how quantitative variables were handled in the analyses. If applicable, describe which groupings were chosen and why	5-6
Statistical methods	12	(*a*) Describe all statistical methods, including those used to control for confounding	5-6
		(*b*) Describe any methods used to examine subgroups and interactions	5-6
		(*c*) Explain how missing data were addressed	5-6
		(*d*) *Cohort study*—If applicable, explain how loss to follow-up was addressed<br>*Case-control study*—If applicable, explain how matching of cases and controls was addressed<br>*Cross-sectional study*—If applicable, describe analytical methods taking account of sampling strategy	N/A
		(*e*) Describe any sensitivity analyses	5-6
**Results**			
Participants	13*	(a) Report numbers of individuals at each stage of study—eg numbers potentially eligible, examined for eligibility, confirmed eligible, included in the study, completing follow-up, and analysed	6, Figure 1, Table 1
		(b) Give reasons for non-participation at each stage	3, Figure 1
		(c) Consider use of a flow diagram	Figure 1

Descriptive data	14*	(a) Give characteristics of study participants (eg demographic, clinical, social) and information on exposures and potential confounders	6, Table 1
		(b) Indicate number of participants with missing data for each variable of interest	Table 1
		(c) *Cohort study*—Summarise follow-up time (eg, average and total amount)	
Outcome data	15*	*Cohort study*—Report numbers of outcome events or summary measures over time	N/A
		Case-control study—Report numbers in each exposure category, or summary measures of exposure	N/A
		Cross-sectional study—Report numbers of outcome events or summary measures	N/A
Main results	16	(*a*) Give unadjusted estimates and, if applicable, confounder-adjusted estimates and their precision (eg, 95% confidence interval). Make clear which confounders were adjusted for and why they were included	11-12, Table 4-5
		(*b*) Report category boundaries when continuous variables were categorized	Table 2-3
		(*c*) If relevant, consider translating estimates of relative risk into absolute risk for a meaningful time period	N/A
Other analyses	17	Report other analyses done—eg analyses of subgroups and interactions, and sensitivity analyses	7-8, Table 2-3
**Discussion**			
Key results	18	Summarise key results with reference to study objectives	14
Limitations	19	Discuss limitations of the study, taking into account sources of potential bias or imprecision. Discuss both direction and magnitude of any potential bias	18
Interpretation	20	Give a cautious overall interpretation of results considering objectives, limitations, multiplicity of analyses, results from similar studies, and other relevant evidence	18
Generalisability	21	Discuss the generalisability (external validity) of the study results	14-18
**Other information**			
Funding	22	Give the source of funding and the role of the funders for the present study and, if applicable, for the original study on which the present article is based	19

Supplementary Table S2. Comparative characteristics of the WHOQOL-BREF, AIS, and SWLS scales.

Scale	Measurement scope	Number of items and scoring	Scale type	Strengths	Limitations
WHOQOL-BREF	Quality of life across physical, psychological, social, and environmental domains	26 items; Likert scale; higher scores indicate better quality of life	Multidimensional, generic	Comprehensive assessment of quality of life across multiple areas of functioning	Longer completion time and less detail within individual domains
AIS	Acceptance of illness and its psychosocial consequences	8 items; score range 8–40; higher scores indicate greater illness acceptance	Unidimensional, health-specific	Focused on illness acceptance; short and easy to interpret	Limited to illness-related aspects without assessing overall quality of life
SWLS	Global life satisfaction	5 items; score range 5–35; higher scores indicate greater satisfaction	Unidimensional, generic	Brief and simple questionnaire measuring overall life satisfaction	Does not analyze specific life domains and does not directly account for health status

Abbreviations: AIS, Acceptance and Action Scale; SWLS, Satisfaction With Life Scale; WHOQOL-BREF, World Health Organization Quality of Life – BREF.

Supplementary Table S3. Domains of the WHOQOL-BREF scale in the entire cohort.

	Whole cohort, N=220		
**Age**	**<65 years old**	**≥65 years old**	**p-value**
WHOQOL-BREF Somatic domain (0-100), Me (1Q-3Q)	60.71 (46.43–67.86)	57.14 (42.86–64.29)	0.319
WHOQOL-BREF Psychological domain (0-100), Me (1Q-3Q)	66.67 (54.17–75.00)	66.67 (54.17–75.00)	0.617
WHOQOL-BREF Social domain (0-100), Me (1Q-3Q)	75.00 (58.33–83.33)	66.67 (50.00–75.00)	<0.001
WHOQOL-BREF Environmental domain (0-100), Me (1Q-3Q)	71.88 (59.38–75.00)	75.00 (56.25–78.12)	0.786
**Sex**	**Female**	**Male**	
WHOQOL-BREF Somatic domain (0-100), Me (1Q-3Q)	60.71 (50.00–67.86)	57.14 (42.86–64.29)	0.027
WHOQOL-BREF Psychological domain (0-100), Me (1Q-3Q)	70.83 (59.38–75.00)	66.67 (54.17–75.00)	0.032
WHOQOL-BREF Social domain (0-100), Me (1Q-3Q)	70.83 (58.33–75.00)	66.67 (50.00–75.00)	0.1
WHOQOL-BREF Environmental domain (0-100), Me (1Q-3Q)	75.00 (68.75–83.59)	71.88 (56.25–75.00)	0.003
**Smoking**	**Non-smokers**	**Current smokers**	
WHOQOL-BREF Somatic domain (0-100), Me (1Q-3Q)	60.71 (46.43–67.86)	55.36 (46.43–64.29)	0.193
WHOQOL-BREF Psychological domain (0-100), Me (1Q-3Q)	75.00 (62.50–79.17)	62.50 (51.04–75.00)	<0.001

WHOQOL-BREF Social domain (0-100), Me (1Q-3Q)	75.00 (58.33–83.33)	66.67 (50.00–75.00)	<0.001
WHOQOL-BREF Environmental domain (0-100), Me (1Q-3Q)	75.00 (68.75–84.38)	67.19 (56.25–75.00)	<0.001
**Alcohol use**	**Non-drinkers**	**Drinkers**	
WHOQOL-BREF Somatic domain (0-100), Me (1Q-3Q)	60.71 (50.00–67.86)	50.00 (39.29–60.71)	0.005
WHOQOL-BREF Psychological domain (0-100), Me (1Q-3Q)	70.83 (58.33–75.00)	54.17 (50.00–75.00)	0.002
WHOQOL-BREF Social domain (0-100), Me (1Q-3Q)	75.00 (58.33–75.00)	58.33 (50.00–75.00)	0.01
WHOQOL-BREF Environmental domain (0-100), Me (1Q-3Q)	75.00 (65.62–78.12)	59.38 (50.00–75.00)	<0.001
**BMI**	**<30 kg/m2**	**≥30 kg/m2**	
WHOQOL-BREF Somatic domain (0-100), Me (1Q-3Q)	60.71 (50.00–67.86)	50.00 (41.96–60.71)	<0.001
WHOQOL-BREF Psychological domain (0-100), Me (1Q-3Q)	66.67 (58.33–75.00)	58.33 (50.00–75.00)	0.005
WHOQOL-BREF Social domain (0-100), Me (1Q-3Q)	75.00 (58.33–75.00)	66.67 (58.33–75.00)	0.133
WHOQOL-BREF Environmental domain (0-100), Me (1Q-3Q)	75.00 (59.38–78.12)	65.62 (53.12–78.12)	0.065
**Previous MI**	**no**	**yes**	
WHOQOL-BREF Somatic domain (0-100), Me (1Q-3Q)	60.71 (46.43-67.86)	50.00 (42.86-60.71)	0.02

WHOQOL-BREF Psychological domain (0-100), Me (1Q-3Q)	66.67 (54.17-75.00)	62.50 (51.04-75.00)	0.205
WHOQOL-BREF Social domain (0-100), Me (1Q-3Q)	75.00 (58.33-75.00)	66.67 (50.00-66.67)	0.026
WHOQOL-BREF Environmental domain (0-100), Me (1Q-3Q)	75.00 (59.38-78.12)	65.62 (56.25-75.00)	0.059
**Hypertension**	**no**	**yes**	
WHOQOL-BREF Somatic domain (0-100), Me (1Q-3Q)	60.71 (53.57-67.86)	53.57 (42.86-64.29)	0.002
WHOQOL-BREF Psychological domain (0-100), Me (1Q-3Q)	75.00 (62.50-81.25)	62.50 (50.00-75.00)	<0.001
WHOQOL-BREF Social domain (0-100), Me (1Q-3Q)	75.00 (66.67-83.33)	66.67 (50.00-75.00)	<0.001
WHOQOL-BREF Environmental domain (0-100), Me (1Q-3Q)	75.00 (71.88-84.38)	68.75 (56.25-75.00)	<0.001
**Heart failure**	**no**	**yes**	
WHOQOL-BREF Somatic domain (0-100), Me (1Q-3Q)	57.14 (46.43-65.18)	53.57 (50.00-60.71)	0.588
WHOQOL-BREF Psychological domain (0-100), Me (1Q-3Q)	66.67 (54.17-75.00)	75.00 (58.33-79.17)	0.308
WHOQOL-BREF Social domain (0-100), Me (1Q-3Q)	66.67 (58.33-75.00)	66.67 (58.33-91.67)	0.379
WHOQOL-BREF Environmental domain (0-100), Me (1Q-3Q)	75.00 (59.38-78.12)	71.88 (65.62-93.75)	0.39
**Diabetes mellitus**	**no**	**yes**	

WHOQOL-BREF Somatic domain (0-100), Me (1Q-3Q)	60.71 (50.00-67.86)	50.00 (35.71-57.14)	<0.001
WHOQOL-BREF Psychological domain (0-100), Me (1Q-3Q)	70.83 (54.17-75.00)	62.50 (54.17-75.00)	0.047
WHOQOL-BREF Social domain (0-100), Me (1Q-3Q)	75.00 (58.33-75.00)	66.67 (50.00-75.00)	0.013
WHOQOL-BREF Environmental domain (0-100), Me (1Q-3Q)	75.00 (59.38-78.12)	68.75 (53.12-78.12)	0.126
**Chronic obstructive pulmonary disease**	**no**	**yes**	
WHOQOL-BREF Somatic domain (0-100), Me (1Q-3Q)	57.14 (46.43-66.96)	50.00 (41.07-55.36)	0.11
WHOQOL-BREF Psychological domain (0-100), Me (1Q-3Q)	66.67 (54.17-75.00)	50.00 (41.67-60.42)	<0.001
WHOQOL-BREF Social domain (0-100), Me (1Q-3Q)	66.67 (58.33-75.00)	58.33 (45.83-70.83)	0.09
WHOQOL-BREF Environmental domain (0-100), Me (1Q-3Q)	75.00 (59.38-78.12)	53.12 (50.00-65.62)	0.002

Abbreviations: BMI, Body Mass Index; Me, Median; MI, Myocardial Infarction; N, Number of subjects; Q, Quartile; WHOQOL-BREF, World Health Organization Quality of Life – BREF.

Supplementary Table S4. Domains of the WHOQOL-BREF scale in subgroups.

Variable	*MI, N=110*			*CCS, N=110*		
**Age**	**<65 years old**	**≥65 years old**	**p-value**	**<65 years old**	**≥65 years old**	**p-value**
WHOQOL-BREF Somatic domain (0-100), Me (1Q-3Q)	60.71 (47.32–64.29)	57.14 (42.86–66.07)	0.388	57.14 (48.21–67.86)	53.57 (50.00–64.29)	0.612
WHOQOL-BREF Psychological domain (0-100), Me (1Q-3Q)	66.67 (54.17–75.00)	70.83 (58.33–79.17)	0.444	70.83 (56.25–75.00)	62.50 (54.17–75.00)	0.161
WHOQOL-BREF Social domain (0-100), Me (1Q-3Q)	75.00 (58.33–75.00)	58.33 (54.17–75.00)	0.071	75.00 (58.33–83.33)	66.67 (50.00–75.00)	0.003
WHOQOL-BREF Environmental domain (0-100), Me (1Q-3Q)	73.44 (59.38–75.00)	75.00 (65.62–81.25)	0.224	71.88 (59.38–75.00)	68.75 (56.25–75.00)	0.587
**Sex**	**Female**	**Male**	**p-value**	**Female**	**Male**	**p-value**
WHOQOL-BREF Somatic domain (0-100), Me (1Q-3Q)	60.71 (50.00–67.86)	57.14 (42.86–64.29)	0.135	60.71 (51.79–67.86)	53.57 (42.86–64.29)	0.104
WHOQOL-BREF Psychological domain (0-100), Me (1Q-3Q)	70.83 (60.42–79.17)	66.67 (54.17–75.00)	0.192	66.67 (60.42–75.00)	62.50 (50.00–75.00)	0.051
WHOQOL-BREF Social domain (0-100), Me (1Q-3Q)	66.67 (58.33–75.00)	70.83 (58.33–75.00)	0.881	75.00 (66.67–75.00)	66.67 (50.00–75.00)	0.011

WHOQOL-BREF Environmental domain (0-100), Me (1Q-3Q)	78.12 (71.88–82.81)	75.00 (59.38–77.34)	0.073	75.00 (67.19–82.81)	65.62 (56.25–75.00)	0.004
**Smoking**	**Non-smokers**	**Current smokers**	**p-value**	**Non-smokers**	**Current smokers**	**p-value**
WHOQOL-BREF Somatic domain (0-100), Me (1Q-3Q)	60.71 (42.86–67.86)	57.14 (46.43–64.29)	0.413	57.14 (46.43–67.86)	53.57 (50.00–64.29)	0.372
WHOQOL-BREF Psychological domain (0-100), Me (1Q-3Q)	75.00 (64.58–79.17)	62.50 (54.17–75.00)	0.004	75.00 (58.33–76.04)	58.33 (50.00–75.00)	0.001
WHOQOL-BREF Social domain (0-100), Me (1Q-3Q)	75.00 (58.33–83.33)	66.67 (50.00–75.00)	0.003	66.67 (58.33–83.33)	66.67 (50.00–75.00)	0.028
WHOQOL-BREF Environmental domain (0-100), Me (1Q-3Q)	75.00 (71.88–84.38)	70.31 (57.03–75.00)	0.003	75.00 (64.84–82.03)	65.62 (55.47–75.00)	0.002
**Alcohol use**	**Non-drinkers**	**Drinkers**	**p-value**	**Non-drinkers**	**Drinkers**	**p-value**
WHOQOL-BREF Somatic domain (0-100), Me (1Q-3Q)	60.71 (46.43–67.86)	50.00 (36.61–57.14)	0.023	57.14 (50.00–67.86)	53.57 (39.29–64.29)	0.085
WHOQOL-BREF Psychological domain (0-100), Me (1Q-3Q)	70.83 (58.33–79.17)	54.17 (42.71–72.92)	0.009	70.83 (54.17–75.00)	58.33 (50.00–70.83)	0.103

WHOQOL-BREF Social domain (0-100), Me (1Q-3Q)	75.00 (58.33–75.00)	62.50 (50.00–75.00)	0.103	66.67 (58.33–75.00)	58.33 (45.83–75.00)	0.044
WHOQOL-BREF Environmental domain (0-100), Me (1Q-3Q)	75.00 (65.62–81.25)	62.50 (50.00–75.00)	0.009	75.00 (59.38–75.00)	56.25 (50.00–73.44)	0.006
**BMI**	**<30 kg/m2**	**≥30 kg/m2**	**p-value**	**<30 kg/m2**	**≥30 kg/m2**	**p-value**
WHOQOL-BREF Somatic domain (0-100), Me (1Q-3Q)	60.71 (46.43–67.86)	50.00 (42.86–59.82)	0.083	57.14 (50.00–67.86)	50.00 (39.29–58.93)	0.001
WHOQOL-BREF Psychological domain (0-100), Me (1Q-3Q)	66.67 (58.33–79.17)	64.58 (51.04–75.00)	0.226	68.75 (58.33–75.00)	56.25 (50.00–69.79)	0.005
WHOQOL-BREF Social domain (0-100), Me (1Q-3Q)	66.67 (58.33–75.00)	70.83 (58.33–81.25)	0.911	75.00 (58.33–75.00)	66.67 (58.33–66.67)	0.037
WHOQOL-BREF Environmental domain (0-100), Me (1Q-3Q)	75.00 (65.62–79.69)	70.31 (57.03–78.12)	0.519	75.00 (59.38–75.00)	64.06 (50.78–75.00)	0.043
**Previous MI**	**no**	**yes**	**p-value**	**no**	**yes**	**p-value**
WHOQOL-BREF Somatic domain (0-100), Me (1Q-3Q)	N/A	N/A	N/A	60.71 (51.79-67.86)	50.00 (42.86-60.71)	0.012
WHOQOL-BREF Psychological domain (0-100), Me (1Q-3Q)	N/A	N/A	N/A	66.67 (54.17-75.00)	62.50 (54.17-75.00)	0.594

WHOQOL-BREF Social domain (0-100), Me (1Q-3Q)	N/A	N/A	N/A	75.00 (58.33-75.00)	66.67 (50.00-66.67)	0.044
WHOQOL-BREF Environmental domain (0-100), Me (1Q-3Q)	N/A	N/A	N/A	75.00 (56.25-75.00)	65.62 (56.25-75.00)	0.292
**Hypertension**	**no**	**yes**	**p-value**	**no**	**yes**	**p-value**
WHOQOL-BREF Somatic domain (0-100), Me (1Q-3Q)	60.71 (57.14-71.43)	53.57 (42.86-64.29)	0.016	60.71 (53.57-67.86)	53.57 (46.43-64.29)	0.049
WHOQOL-BREF Psychological domain (0-100), Me (1Q-3Q)	75 (66.67-83.33)	66.67 (52.08-75)	<0.001	75.00 (62.50-79.17)	62.50 (50.00-75.00)	0.005
WHOQOL-BREF Social domain (0-100), Me (1Q-3Q)	75.00 (75.00-83.33)	58.33 (50.00-75.00)	<0.001	75.00 (66.67-83.33)	66.67 (50.00-75.00)	<0.001
WHOQOL-BREF Environmental domain (0-100), Me (1Q-3Q)	75.00 (72.66-81.25)	71.88 (54.69-78.12)	0.002	75.00 (65.62-84.38)	68.75 (56.25-75.00)	0.004
**Heart failure**	**no**	**yes**	**p-value**	**no**	**yes**	**p-value**
WHOQOL-BREF Somatic domain (0-100), Me (1Q-3Q)	N/A	N/A	N/A	57.14 (49.11-65.18)	53.57 (47.32-59.82)	0.487
WHOQOL-BREF Psychological domain (0-100), Me (1Q-3Q)	N/A	N/A	N/A	66.67 (54.17-75.00)	70.83 (57.29-79.17)	0.404

WHOQOL-BREF Social domain (0-100), Me (1Q-3Q)	N/A	N/A	N/A	66.67 (56.25-75.00)	62.50 (58.33-85.42)	0.672
WHOQOL-BREF Environmental domain (0-100), Me (1Q-3Q)	N/A	N/A	N/A	68.75 (56.25-75.00)	70.31 (63.28-86.72)	0.538
**Diabetes mellitus**	**no**	**yes**	**p-value**	**no**	**yes**	**p-value**
WHOQOL-BREF Somatic domain (0-100), Me (1Q-3Q)	60.71 (50.00-67.86)	46.43 (37.50-55.36)	0.001	57.14 (50-67.86)	53.57 (37.5-58.93)	0.006
WHOQOL-BREF Psychological domain (0-100), Me (1Q-3Q)	70.83 (58.33-75)	62.5 (55.21-79.12)	0.136	66.67 (54.17-75.00)	58.33 (52.08-72.92)	0.125
WHOQOL-BREF Social domain (0-100), Me (1Q-3Q)	75.00 (58.33-75.00)	58.33 (52.08-72.92)	0.097	66.67 (58.33-75.00)	66.67 (54.17-70.83)	0.087
WHOQOL-BREF Environmental domain (0-100), Me (1Q-3Q)	75.00 (65.62-78.12)	71.88 (54.69-81.25)	0.632	75.00 (59.38-75.00)	65.62 (54.69-75.00)	0.105
**Chronic obstructive pulmonary disease**	**no**	**yes**	**p-value**	**no**	**yes**	**p-value**
WHOQOL-BREF Somatic domain (0-100), Me (1Q-3Q)	60.71 (44.64-66.07)	50.00 (44.64-55.36)	0.34	57.14 (50.00-66.07)	50.00 (39.29-53.57)	0.199

WHOQOL-BREF Psychological domain (0-100), Me (1Q-3Q)	70.83 (58.33-79.17)	54.17 (46.88-64.58)	0.056	66.67 (54.17-75.00)	45.83 (29.17-54.17)	0.007
WHOQOL-BREF Social domain (0-100), Me (1Q-3Q)	66.67 (58.33-75.00)	70.83 (54.17-75.00)	0.93	66.67 (58.33-75.00)	50.00 (41.67-58.33)	0.011
WHOQOL-BREF Environmental domain (0-100), Me (1Q-3Q)	75.00 (65.62-81.25)	56.25 (50.78-68.75)	0.034	71.88 (56.25-75.00)	50.00 (50.00-65.62)	0.02

Abbreviations: BMI, Body Mass Index; Me, Median; MI, Myocardial Infarction; N, Number of subjects; Q, Quartile; WHOQOL-BREF, World Health Organization Quality of Life – BREF.
